# Learned Vocal Variation Is Associated with Abrupt Cryptic Genetic Change in a Parrot Species Complex

**DOI:** 10.1371/journal.pone.0050484

**Published:** 2012-12-05

**Authors:** Raoul F. H. Ribot, Katherine L. Buchanan, John A. Endler, Leo Joseph, Andrew T. D. Bennett, Mathew L. Berg

**Affiliations:** 1 Centre for Integrative Ecology, School of Life and Environmental Sciences, Deakin University, Geelong, Victoria, Australia; 2 Centre for Behavioural Biology, School of Biological Sciences, University of Bristol, Bristol, United Kingdom; 3 Australian National Wildlife Collection, Commonwealth Scientific and Industrial Research Organisation Ecosystem Sciences, Canberra, Australia; 4 School of Earth and Environmental Sciences, University of Adelaide, Adelaide, South Australia, Australia; University of Bristol, United Kingdom

## Abstract

Contact zones between subspecies or closely related species offer valuable insights into speciation processes. A typical feature of such zones is the presence of clinal variation in multiple traits. The nature of these traits and the concordance among clines are expected to influence whether and how quickly speciation will proceed. Learned signals, such as vocalizations in species having vocal learning (e.g. humans, many birds, bats and cetaceans), can exhibit rapid change and may accelerate reproductive isolation between populations. Therefore, particularly strong concordance among clines in learned signals and population genetic structure may be expected, even among continuous populations in the early stages of speciation. However, empirical evidence for this pattern is often limited because differences in vocalisations between populations are driven by habitat differences or have evolved in allopatry. We tested for this pattern in a unique system where we may be able to separate effects of habitat and evolutionary history. We studied geographic variation in the vocalizations of the crimson rosella (*Platycercus elegans*) parrot species complex. Parrots are well known for their life-long vocal learning and cognitive abilities. We analysed contact calls across a *ca* 1300 km transect encompassing populations that differed in neutral genetic markers and plumage colour. We found steep clinal changes in two acoustic variables (fundamental frequency and peak frequency position). The positions of the two clines in vocal traits were concordant with a steep cline in microsatellite-based genetic variation, but were discordant with the steep clines in mtDNA, plumage and habitat. Our study provides new evidence that vocal variation, in a species with vocal learning, can coincide with areas of restricted gene flow across geographically continuous populations. Our results suggest that traits that evolve culturally can be strongly associated with reduced gene flow between populations, and therefore may promote speciation, even in the absence of other barriers.

## Introduction

A fundamental property of contact zones between subspecies or closely related species is the presence of stable and concordant steep clines in multiple traits. This can arise either from secondary contact of formerly isolated divergent populations or environmentally induced evolutionary divergence along environmental gradients, and can be a stage in the process of speciation [1], [2], [3]. The clines will not remain concordant for long unless the traits are genetically or functionally linked, or if there is a partial barrier to gene flow at or near the centre of the clines, or if the same environmental gradient affects all traits equally [1], [3], [4], [5]. Cline concordance is more likely to be maintained, and the probability of speciation increased, if some of the concordant clines involve traits associated with mate choice and breeding. For example, if vocal signals are used in mate choice, and if vocal traits form steep clines, the individuals from either side of the cline may be less likely to mate with each other if they were to disperse into each other’s ranges [3], [6], [7], [8]. Such partial isolation would be expected to steepen the vocal clines and simultaneously promote divergence of other traits, making speciation more likely [1], [5].

Divergence between populations may be more rapid if such signals are learned rather than genetically determined [3], [7]. Therefore, we expect strong divergence in learned signals relative to other signals, particularly during the early stages of speciation. In several taxa, including higher primates, cetaceans, bats and many birds, vocalizations are learned and are important mating signals [6], [9], [10], [11], [12], [13]. A major prediction is strong concordance between vocal clines and reduced gene flow in such taxa. In humans there are positive associations between vocal signals and genetic variation between populations (e.g. [14], [15], [16], [17]), but there is very limited empirical evidence for concordance between learned vocal variation and population genetic structure in the three groups of birds that display vocal learning [6], [18]: oscines [19], [20], [21], [22], parrots [23], [24] and hummingbirds (no studies to our knowledge). Furthermore, results are often confounded by the influences of habitat, morphology, evolutionary history, or a low level of population genetic structure. Here we report on a parrot species complex in which vocal variation and microsatellite-inferred genetic clines are concordant with each other but not with known plumage, mitochondrial DNA or habitat clines.

**Figure 1:**
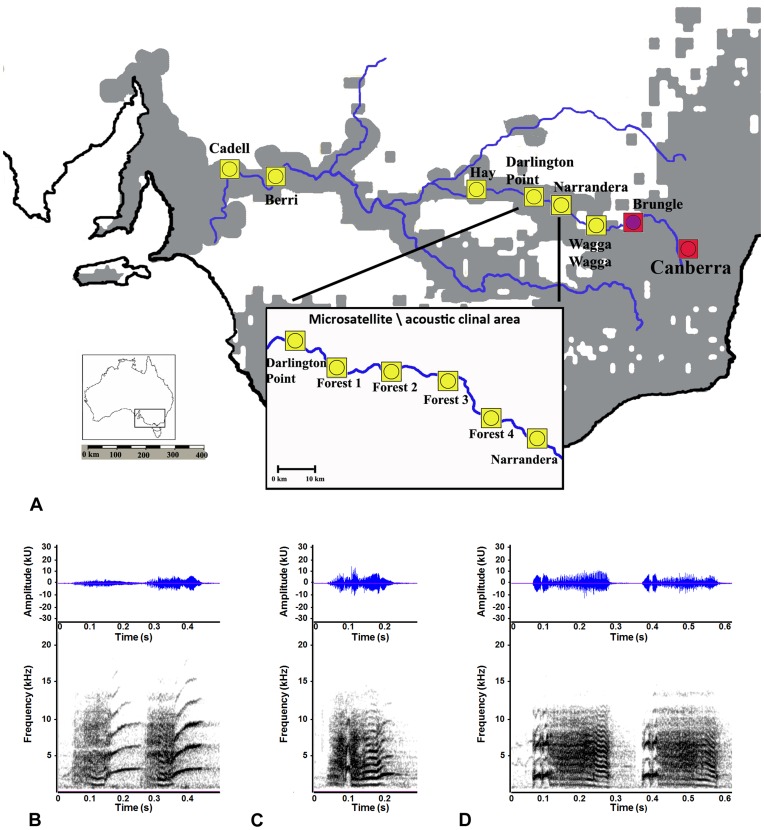
Overview of study sites and contact call variation. (a) Distribution of *Platycercus elegans* (grey overlay) showing recording sites. The yellow squares are recording sites with yellow phenotype birds; red squares are recording sites with crimson phenotype birds. The dots inside the squares indicate the mtDNA of the population: yellow dots = yellow subspecies (*P. e. flaveolus*), purple spots = hybrid Western Slopes population, red dots = crimson subspecies (*P. e. elegans*). The cut out gives a more detailed look of the six recording sites where the clines in microsatellite markers occur. The blue line denotes the Murray-Murrumbidgee river system. (b) Spectrogram with waveform of two contact calls from Berri (in the *P. e. flaveolus* distribution). (c) Spectrogram with waveform of a contact call from the clinal microsatellite area. (d) Spectrogram with waveform of two contact calls from Brungle (in the hybrid Western Slopes population).

Studies of several parrot species to date confirm that their calls are learned throughout their lifetime and fulfil a variety of social functions [13], [25], [26]. Evidence from several species has demonstrated that parrot contact calls are used in mate choice, pair bonding and mate recognition [26], [27], [28], [29], [30], [31]. Australian parrots in the crimson rosella complex (*Platycercus elegans*) form steep plumage colour and genetic clines, however these clines are discordant [32], [33], [34]. This mismatch occurs close to the area where two phenotypically divergent subspecies, the predominantly red crimson rosella (*P. e. elegans*) and the contrastingly yellow rosella (*P. e. flaveolus*), meet in riparian habitats along a major river system and form a narrow hybrid zone [33]; Fig. 1). To the west of this zone for several hundred kilometres, yellow rosellas occur in riparian open woodlands surrounded by grasslands and low open woodlands, whereas to the zone’s east crimson birds essentially inhabit dense woodlands and forests. However, according to our recent microsatellite analysis, yellow birds up to *ca* 200 km west of the hybrid zone were consistently more similar genetically to crimson populations elsewhere in south-eastern Australia [33].

We intensively sampled these closely related but genetically divergent populations of *P. elegans* for vocal characters in and on either side of the area where we have previously identified discordance between microsatellite and phenotypic diversity [33]. Our broad aim was to provide insight into the relationships between culturally transmitted and genetic traits, and the role that learned signals may play in population divergence and incipient speciation. A more specific aim was to test whether clinal variation and divergence in vocal characters correlate more strongly with known mitochondrial DNA (mtDNA), plumage and habitat clines, or with previously documented, cryptic population genetic structure in *P. elegans*.

## Materials and Methods

### Ethics Statement

This study conformed to the laws for animal experimentation in Australia (Deakin University Animal Ethics Committee approval A33/2008; New South Wales National Parks and Wildlife Service Licence Number S11436 and South Australian Department for Environment and Heritage Permit Number Y24784), and was carried out on privately owned properties with permission.

### Study Sites

We recorded 182 free-living individuals from 12 sites along the Murray-Murrumbidgee river system in Australia (Fig. 1). These sites spanned over 1200 km of continuous habitat from Cadell (South Australia), in the western part of the range of the yellow rosella subspecies (*P. e. flaveolus*), to Canberra (Australian Capital Territory), which lies within the range of the crimson rosella subspecies (*P. e. elegans*). These sites encompassed two genetic clusters identified by a previous Geneland analysis of 14 microsatellite loci where they were termed Central^GL^ and Eastern^GL^ and two mtDNA (ND2) haplotype groups in earlier work by our group [33] (shown in Fig. 1). The difference in microsatellite markers between individuals at different recording sites was confirmed with an analysis using STRUCTURE [35]. The average distance between adjacent recording sites was >10 km to increase independence between recording sites and to minimize the chance of unknowingly including the same individuals in the analysis more than once [36].

### Recording and Acoustic Analyses

We analyzed the *P. elegans* contact call, which is the most common call produced by rosellas and is highly variable (Figure 1; [36]). Contact calls are commonly studied in parrot communication, and are thought to facilitate a range of social activities and be involved mate preferences (e.g. [25], [26], [27], [28], [29], [30], [31], [37], [38], [39]). We analyzed 556 contact calls recorded from 182 individuals. Sites were visited over the period 2004–2009, and recordings were made during daylight hours (0700–2000 h) in December and January using the methods described in earlier work by our group [36], [40].

Sound files (uncompressed wav format, sampling rate 44.1 kHz, 16-bit) were analyzed with Raven Pro 1.4 (Cornell Lab of Ornithology, New York, USA) and Sound Analysis Pro (SAP) 1.04 [41]. Calls were processed and analyzed in random order and blindly with respect to their population of origin. Up to four clear calls per individual (3.05±1.12) were chosen on the basis of amplitude and background noise, and band pass filtered using Raven to remove noise <0.5kHz. Spectrograms and waveforms of the calls were produced using Raven (windows setting: Blackman; size: 400 samples; DFT size: 2048 samples; overlap: 90%) and Sound Analysis Pro (standard settings; FFT window  =  1024 samples; overlap  =  85%; spectrum range  =  18 kHz). For each contact call we measured five acoustic variables that reflect temporal and structural properties of the call [36]. These are: (1) call duration, measured in Raven using the waveform, (2) peak frequency, the frequency with greatest amplitude as measured automatically in Raven, (3) fundamental frequency, the fundamental frequency of harmonically rich notes that formed the longest part of the call measured manually in Raven, (4) mean frequency modulation (FM), measured automatically in SAP, which indicates the deviation of frequencies in the call (in degrees) in comparison to a constant frequency, and (5) peak frequency pattern, which is the temporal location of the peak frequency within each call, measured automatically in Raven, and presented as a percentage of the total duration of the call. We used an individual mean for each of the five acoustic variables in statistical analyses. Information on intra-individual differences between calls is available in Ribot et al. (2009).

### Statistical Analyses

The five acoustic variables were tested separately, as combining the five acoustic variables using a principal component (PC) analysis would only allow for a small reduction in variables (PC 1 explained 32.9% of the variation, PC 2 explained 24.3%, PC 3 explained 20.3%) and the pairwise correlation coefficients between the acoustic variables were typically low (0.033< *r* <0.378). *P*-values are two-tailed and results were considered significant when *P*<0.05.

We used the cline fitting software C-fit [42] to analyze the acoustic and microsatellite clines. We used the vocalization data directly. For the microsatellite data we used STRUCTURE to estimate the probability of inferred ancestry for each individual, with parameters k = 2 (2 groups assumed), 74 individuals, 15 loci, 100000 burn-in period, 100000 replications, LOCPRIOR model (longitudes). The overall proportion of membership in the two inferred clusters was 0.48 and 0.52, with an estimated allele frequency divergence among populations of 0.0315, and r = 0.86. The probability of each individual being in group 1 formed the cline we analyzed with C-fit (Figure 1). C-fit fits cline models to data assuming a unimodal (one species), bimodal (two species with little or no hybridization) or a trimodal (two species or semispecies with hybridization) model. Models can either be fit to single trait clines, or simultaneously to two or more clines. The Akaike Information Criterion (AIC) is then used to decide which is the best fitting model [42]. We fit clines to the acoustic variables and the microsatellite clines separately, to test which modal model fitted these traits best. We tested the two vocal traits with steep clines jointly for comparison with the sum of the AIC of the single-fitted traits to test for concordance of the two vocal traits [42]. We also tested for concordance between the microsatellite and two steep vocal trait clines.

Mantel tests, implemented in IBD 1.52 (1000 randomizations; [43]), were used to analyze acoustic distances versus geographic distances between the 12 recording sites. For call distances, we calculated Euclidean distances between the recording sites using the means of each acoustic variable for each site. Geographic distances between recording sites were calculated following the major path of the river and suitable habitat (1281 km).

## Results

### Call Structure

In most cases, contact calls consisted of harmonically rich notes (92%, *n* = 514), with the fundamental frequency, where measured, typically falling into one of two frequency ranges, namely 0.7–1.0 kHz or 2.0–3.5 kHz (95%, *n* = 489).

### Acoustic Variation Across the Microsatellite Contact Zone

**Figure 2:**
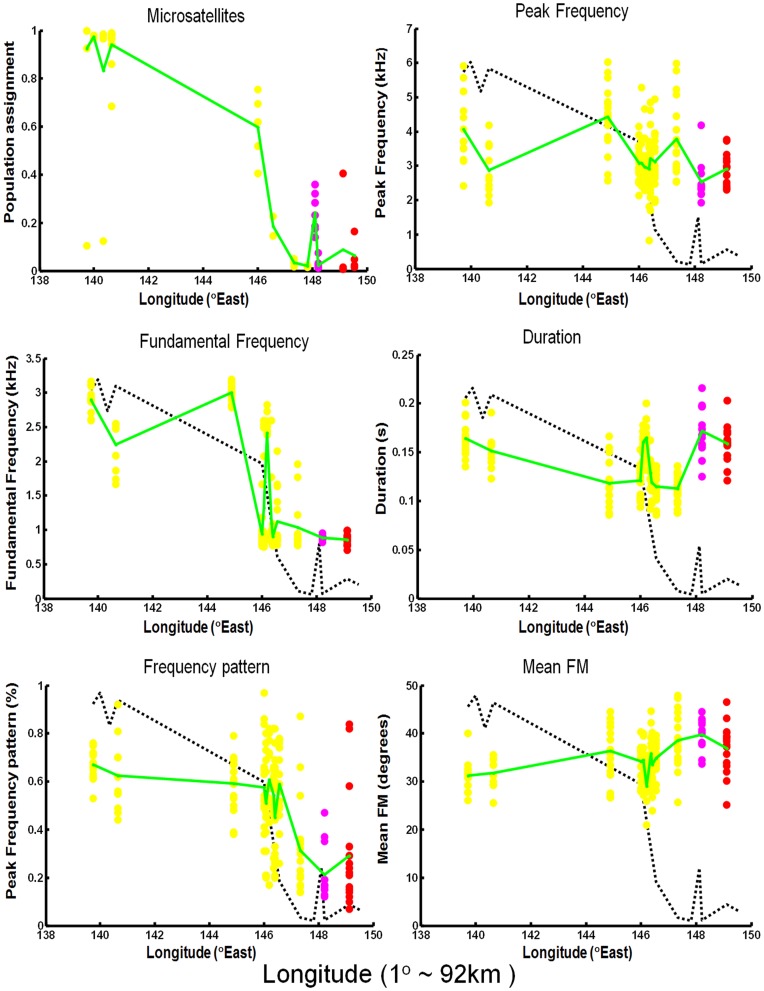
Plots of the microsatellite population assignment (using STRUCTURE) and all five acoustic variables clines. Dots are the mean values for each individual. The three plumage forms/subspecies are indicated by dot colour as follows: *P. e. flaveolus*, yellow dots; the Western Slopes population, purple dots; *P. e. elegans*, red dots. Green lines connect the means of each population, and the dotted line within the vocalization plots indicates the microsatellite cline for comparison. At this latitude 1 degree of longitude is roughly 92km, and the transect changes little with latitude (Fig. 1).

Fundamental frequency, mean frequency modulation (FM) and peak frequency pattern varied clinally across the transect (Fig. 2). Fundamental frequency and peak frequency pattern formed steep clines which were roughly concordant with the steep microsatellite cline (Fig. 2): C-fit analysis [42] showed that, although the two steep vocal trait clines and the microsatellite clines were roughly concordant in position and highly discordant with plumage and mtDNA, their fitted midpoints were still significantly different (Table 1). The position order is (from West to East): fundamental frequency, microsatellite score and peak frequency pattern, a gap, mtDNA haplotype group, and then plumage coloration (Fig. 2).

**Table 1:** Tests for fit of unimodal, bimodal and trimodal cline models for each variable (*A*), tests for common position of two vocalization traits with steep clines (*B*), and tests for common position of the vocalization clines and the microsatellite cline (*C*).

Test	Variable(s)	Model	df	AIC
(*A*) Clines of single traits	Fundamental frequency	Unimodal	7	209.34
		Bimodal	8	−23.3
		Trimodal	14	−52.36*
	Peak frequency pattern	Unimodal	7	−111.94
		Bimodal	8	−145.63*
		Trimodal	11	−145.13*
	Duration	Unimodal	7	−691.09
		Bimodal	8	−785.66*
		Trimodal	14	−784.53*
	Peak frequency	Unimodal	7	469.55
		Bimodal	8	462.59*
		Trimodal	14	487.73
	Mean frequency modulation	Unimodal	7	1117.03
		Bimodal	8	1112.7*
		Trimodal	14	1119.74
	Microsatellite assignment	Unimodal	6	−59.6
		Bimodal	8	−143.91*
		Trimodal	14	−136.2
		Trimodal (hybrid deficiency in east)	11	−204.2*
(*B*) Common position of vocal clines	Fundamental frequency and Peak frequency pattern	Unimodal, Common	7	668.47
		Unimodal, Common position only	13	113.7
		Unimodal, Common position+slope	12	113.42
		Bimodal, Common	8	431.82
		Bimodal, Common position only	16	−120.77
		Bimodal, Common position+slope	15	−133.86
		Trimodal, Common	14	417.38
		Trimodal, Common position only	27	−139.39
		Trimodal, Common position+slope	26	−135.66
	Sum of AIC for vocal traits fitted singly	Trimodal		−197.49*
(*C*) Common position of vocal and microsatellite clines	Peak frequency pattern and microsatellite assignment	Trimodal, Joint fits	14	−194.59
	Peak frequency pattern+microsatellite assignment	Sums of single trait fits		−281.7*
	Fundamental frequency and microsatellite assignment	Trimodal, Joint fits	14	281.41
	Fundamental frequency+microsatellite assignment	Sums of single trait fits		−188.56*

For clines of single traits (*A*), unimodal models were never the best fits. A trimodal model fit best for fundamental frequency, a bimodal model fit best for peak frequency and mean FM, bimodal and trimodal models fit equally well for peak frequency pattern and duration. For microsatellite assignment, a bimodal model fit best but a trimodal model with a deficiency of hybrids in the east was better still.

For tests of common position of the vocal clines (*B*), individual models fit better than pooled models. A trimodal model with an assumption of common position was the best joint fit.

For tests of common position of vocal clines and the microsatellite cline (*C*), individual models fit better than joint models.

* indicates best fitting models.

C-fit analysis of both the vocalization and microsatellite clines (Table 1) showed that unimodal models never fit as well as the alternative bimodal and/or trimodal models, indicating that some form of hybridization is occurring, rather than these traits forming simple clines. The trimodal models fit best for fundamental frequency, the trimodal and bimodal models fit equally well for peak frequency pattern and duration, and the bimodal model had the best fit for peak frequency and mean FM. Unlike the vocal traits, the best fitting models for microsatellites depended upon more assumptions. The best fitting model for microsatellites was trimodal when we constrained the model [42] to have deficiency of hybrids in the east. This model fits better than any other model, including deficiencies in the middle or west, or no deficiencies (Table 1; Fig. S1 and S2). Trimodal models suggest that backcrossing is limited and the populations are partially isolated [42].

**Figure 3:**
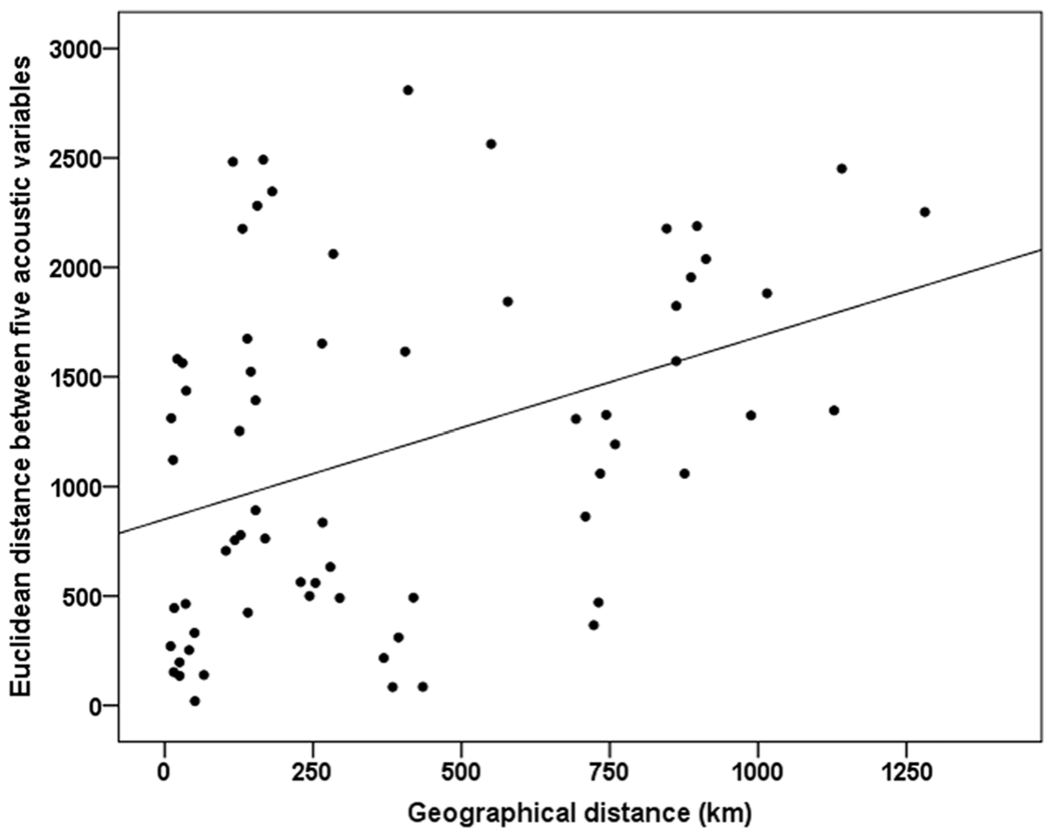
Results from a Mantel test analyzing the relationship between vocal variation (Euclidean distances based on the five acoustic variables between all the recording sites) and geographic distances between recording sites (R^2^ = 0.143; p = 0.036).

A Mantel test using all five acoustic parameters at each site provided evidence for significant isolation-by-distance across the transect (Fig. 3). This test is based on non- straight line (i.e. river-course) geographic distance between recording sites; similar results were obtained when using straight line geographic distances along the river (*r* = 0.41, *P* = 0.025). Mantel tests conducted on each acoustic variable separately (Fig. S3) indicated a pattern of isolation-by-distance which occurred in fundamental frequency and time of peak frequency in call (*r*>0.337, *P*<0.05), and possibly mean FM (*r* = 0.338, *P*<0.1).

## Discussion

In this study we showed that learned vocal variation in *P. elegans* is spatially correlated with microsatellite-based genetic population structure far more strongly than with plumage coloration and mtDNA diversity over a 1300 km transect [33]. To our knowledge, this is the first demonstration of this pattern. Moreover, our study reveals some of the clearest evidence to date for a relationship between presumably neutral genetic variation and vocal variation in any species that displays vocal learning. This supports the notion that vocal learning of birds may play an important role in speciation [20], [44], [45], [46], [47].

C-fit allows identification of clines, tests of concordance, and comparison of unimodal, bimodal and trimodal models [42]. Based on our C-fit analysis, we confidently reject the unimodal scenario. This is because, for all vocal traits considered and for the microsatellite-inferred genetic cline, the bimodal, and in some cases the trimodal, models provided clearly better fits to the data (Table 1). The better fit with the bimodal and/or trimodal models shown in this study suggests that the contact zone, rather than forming simple clines, has two or three sets of genotypes: parental types and hybrids [42]. This is what one would expect during incipient speciation. A trimodal model would best fit the microsatellite genotypes as in the vocal traits where hybrids are starting to become distinct rather than being lost in a continuum of intergrades. At a slightly later stage of speciation reproductive isolation would increase and reduce the incidence of hybrids, leading to the bimodal model being a better fit than the trimodal model. The microsatellites appear to be somewhere between the two with a good fit to either the bimodal or trimodal model and a deficiency to the east. The latter may result from interaction with the other two clines to the east.

There are at least two possible explanations for steep vocal trait clines being roughly congruent with the steep, presumably neutral microsatellite cline, but very discordant with both plumage coloration and mtDNA variation. The simplest hypothesis is that secondary contact occurred between eastern and western populations and that the vocalization and microsatellite clines occur roughly where the contact first occurred. This could result in unimodal, bimodal or trimodal models fitting best depending on the extent of gene flow between the formerly isolated populations. The clines in plumage coloration and mtDNA may have moved eastward until they reached the present transition between open riparian woodland and higher elevation forest [48], [49], [50]; different visual backgrounds then potentially would have caused geographical selection gradients balanced by gene flow and a temporarily moving cline which stopped at the centre of the habitat gradient. This would constitute habitat matching (8, 16–19) for plumage. Environmentally dependent variation in colour vision [51], [52] may also contribute to plumage colour variation due to sensory drive. In addition, mtDNA clines are often displaced relative to nuclear-based clines due to sex-related differences in dispersal or introgression [53], [54]. Under this scenario of a moving hybrid zone for plumage coloration, the microsatellite and vocal clines remain as a “ghost of introgression past” [50]. Unlike *P. elegans* colour variation, habitat matching (8, 16–19) is unlikely to explain the vocal clines because acoustic variation was not concordant with known habitat or topographical variation: steep environmental gradients occur much further east of the vocalization and microsatellite clines, where crimson and yellow birds currently meet [48], [49].

A second hypothesis is that the vocal traits differentiated at random and that this promoted pre-zygotic reproductive isolation arising from mate choice, which is among the functions demonstrated for parrot contact calls [26], [27], [29], [30]. This would subsequently promote differentiation in the microsatellites and other traits on either side of the cline. If the other traits were involved in genetic compatibility then this would accelerate concordant differentiation in both sets of traits [7]. This would most likely result in the bimodal or trimodal models fitting best, because reproductive isolation has already started to occur. The colour traits may have already responded to the environmental gradient further east, although this does not explain the location of mtDNA divergence. It is invalid to assume that different kinds of traits necessarily respond to hybridization in the same way [55]. Based on our present data, it is difficult to distinguish these two hypotheses because it depends on the level of reproductive isolation that has evolved. Importantly, our analysis of vocal and microsatellite clines suggests that the clines signal an early step in the process of speciation before complete reproductive isolation has occurred; this would be true regardless of the extent to which either of the two historical alternatives outlined above may apply.

To date, the studies by Wright et al. [23], [24] on yellow-naped amazon parrots (*Amazona auropalliata*) are among the most comprehensive in examining associations between avian vocal variation and genetic population structure. However, these studies did not find any evidence that geographic variation in contact calls [38] was related to genetic differences between populations, measured with mtDNA [23] and microsatellites [24]. While they detected very little neutral genetic variation between populations using microsatellite data, Wright et al. [24] suggested that the most likely reason for these results is because parrots are open-ended vocal learners and can learn the dialects of new populations after they disperse. A study of mountain white-crowned sparrows [44] indicated that acoustic differences between populations explained a significant amount of microsatellite variation between populations. However, the amount of genetic variation associated with dialects was extremely small (<1%) and much lower than that explained by variation among individuals within recording sites (>98%). Further, a subsequent study by Soha et al. [19] in another subspecies of white-crowned sparrows did not detect a significant relationship between population genetic structure and acoustics at all. They suggested that this could be due to recent population divergence, as they also observed little genetic differentiation overall [19].

A likely explanation for the lack of concordance between dialects and population genetic structure reported in these and other previous studies [19], [20], [21], [22], [23], [24], is that most studies have examined populations with clear vocal dialects but little or no population genetic structure, or used too few or inappropriate molecular markers to detect existing genetic variation. Thus, most previous studies set out to test whether existing dialect boundaries limit gene flow and thus promote genetic differentiation. In some cases, an insufficient amount of time may have passed since the formation of dialects for genetic differences to become evident (e.g. [19], [20]). Learned avian acoustic signals have the potential to change rapidly in birds [6], [7], [56], [57], so markers with relatively rapid mutation rates are more likely to correspond with recent divergence in acoustic signals and events in recent population history. Investigating vocal variation in a species with previously observed distinct genetic variation between populations, as we have done in this study, may provide a more reliable way of testing whether vocal variation and genetic differences are linked.

Furthermore, habitat can determine which acoustic features of vocalizations will be favoured [58], [59], either directly through its effect on which types of sounds are transmitted the best, or indirectly through selection on the morphology of sound-producing structures [60]. Most studies so far have concluded that the social relations between individuals and habitat-dependent selection are more important factors affecting vocal divergence between populations than population genetic structure [10]. However, acoustic traits that are less susceptible to ecological change (e.g. those that are less affected by habitat structure, those with a stronger genetic basis and those more dependent on syringeal structure; [61]) could still be expected to show an association with genetic structure.

### Conclusions

The hypothesis that learned signals, such as many avian and mammal vocalizations, play a role in promoting population divergence and ultimately speciation remains contentious, and to date clear empirical evidence is still largely lacking. A key prediction of this hypothesis, however, is the concordant geographical patterns of signal variation and gene flow in the early stages of speciation. In this study we show that in *P. elegans*, rapid geographical change in contact calls coincides with a steep, microsatellite-inferred (and presumably neutral) genetic cline across geographically continuous populations. Furthermore, these clines occur *ca* 100 km west of steep clines in mtDNA, plumage coloration and habitat. Our study provides new evidence that vocal variation, in a species with vocal learning, coincides with zones of relatively reduced gene flow even in the absence of barriers associated with morphological, habitat or mtDNA differences between populations. Our findings suggest that traits that may rapidly evolve culturally can be strongly associated with recent genetic divergence between populations, and therefore support the hypothesis that such traits can play a key role in promoting speciation. Whether these differences in calls are being used by individuals to discriminate between populations in the area where the genetic discontinuity was identified is unknown, and will require further research.

## Supporting Information

Figure S1 **Histograms displaying the frequency distribution of values of all five acoustic variables for the four sites (Forest 1–4) comprising the area where clines in microsatellite markers and acoustic variation were observed.** Frequencies represent the number of calls.
(TIF)

Figure S2 **Histograms displaying the frequency distribution of values of the five acoustic variables separately for the four recording sites (Forest 1–4) comprising the area where clines in microsatellite markers and acoustic variation were observed.** Each graph consists of four separate windows with histograms, each window displays the values for one recording site. Frequencies represent the number of calls.
(TIF)

Figure S3 **Plots of Euclidian distances between five acoustic variables derived from rosella contact calls versus geographic distance along the path of the river.** The acoustic variables shown are (a) call duration, (b) peak frequency, (c) fundamental frequency, (d) mean frequency modulation, and (e) peak frequency pattern. The Euclidian distance for each acoustic variable is the absolute difference between each recording site in the average value of that acoustic variable. Circles represent comparisons among the four recording sites within each microsatellite group, and bi-coloured squares represent comparisons between recording sites in different microsatellite groups. Colours indicate the microsatellite groups involved in each comparison: Central vs. Central (yellow circles), microsatellite contact zone vs. microsatellite contact zone (purple circles), Eastern vs. Eastern (red circles), Central vs. Eastern (yellow and red squares), Central vs. microsatellite contact zone (yellow and purple squares), Eastern vs. microsatellite contact zone (red and purple squares). Mantel tests indicate significant or near-significant isolation-by-distance in fundamental frequency (*r* = 0.416, P = 0.033), peak frequency pattern (*r* = 0.338, P = 0.047), and mean frequency modulation (*r* = 0.338, P = 0.087), but not call duration (*r* = 0.010, P = 0.406) or peak frequency (*r* = 0.188, P = 0.173).
(TIF)
