# Supplementary figures and images for: Learned Vocal Variation Is Associated with Abrupt Cryptic Genetic Change in a Parrot Species Complex
Source: PLoS One. 2012 Dec 5;7(12):e50484. doi: 10.1371/journal.pone.0050484 (PMC3515628; doi:10.1371/journal.pone.0050484)

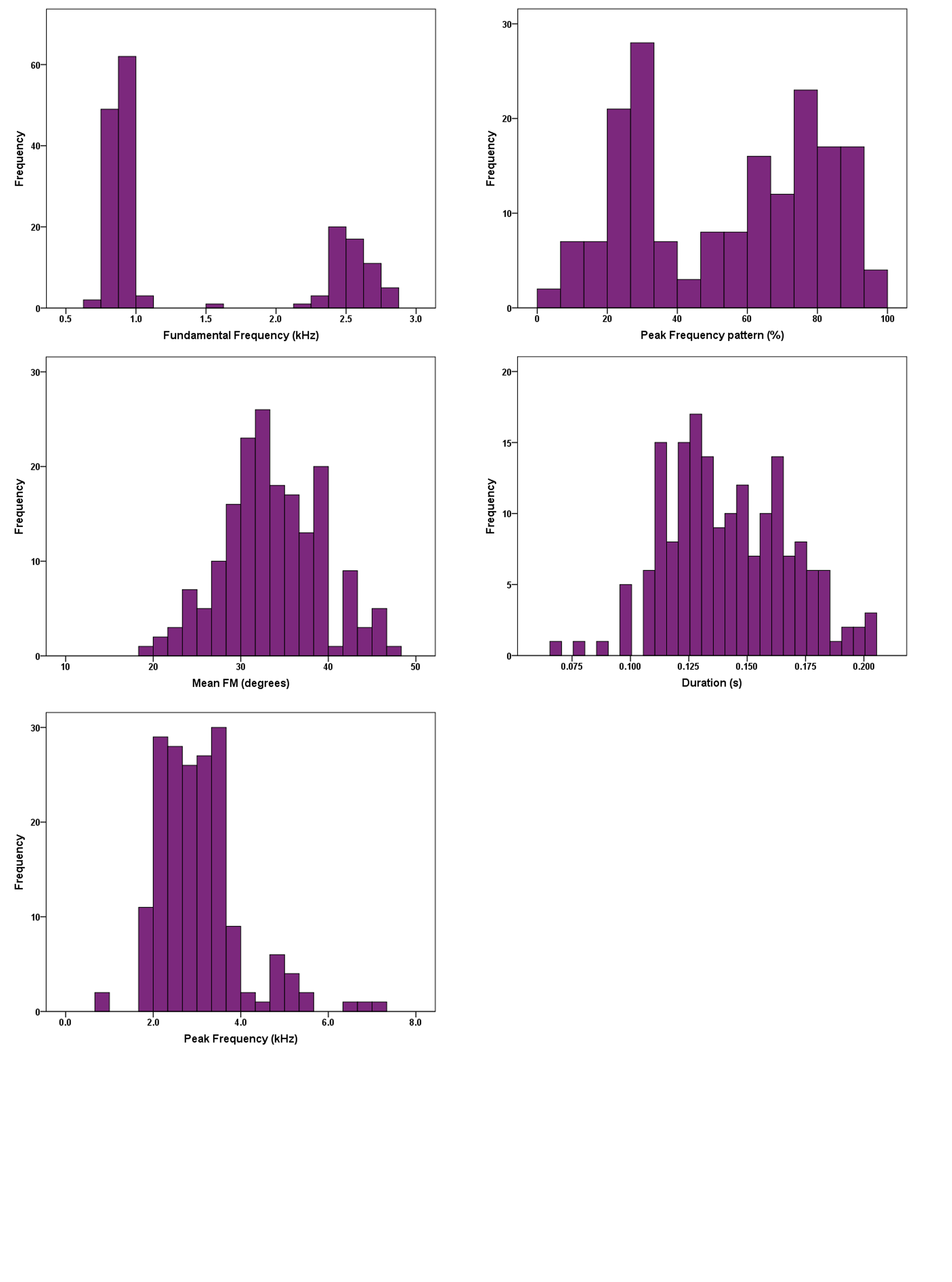

Supplement: Figure S1 — Histograms displaying the frequency distribution of values of all five acoustic variables for the four sites (Forest 1–4) comprising the area where clines in microsatellite markers and acoustic variation were observed. Frequencies represent the number of calls. (TIF) [file pone.0050484.s001.tif]

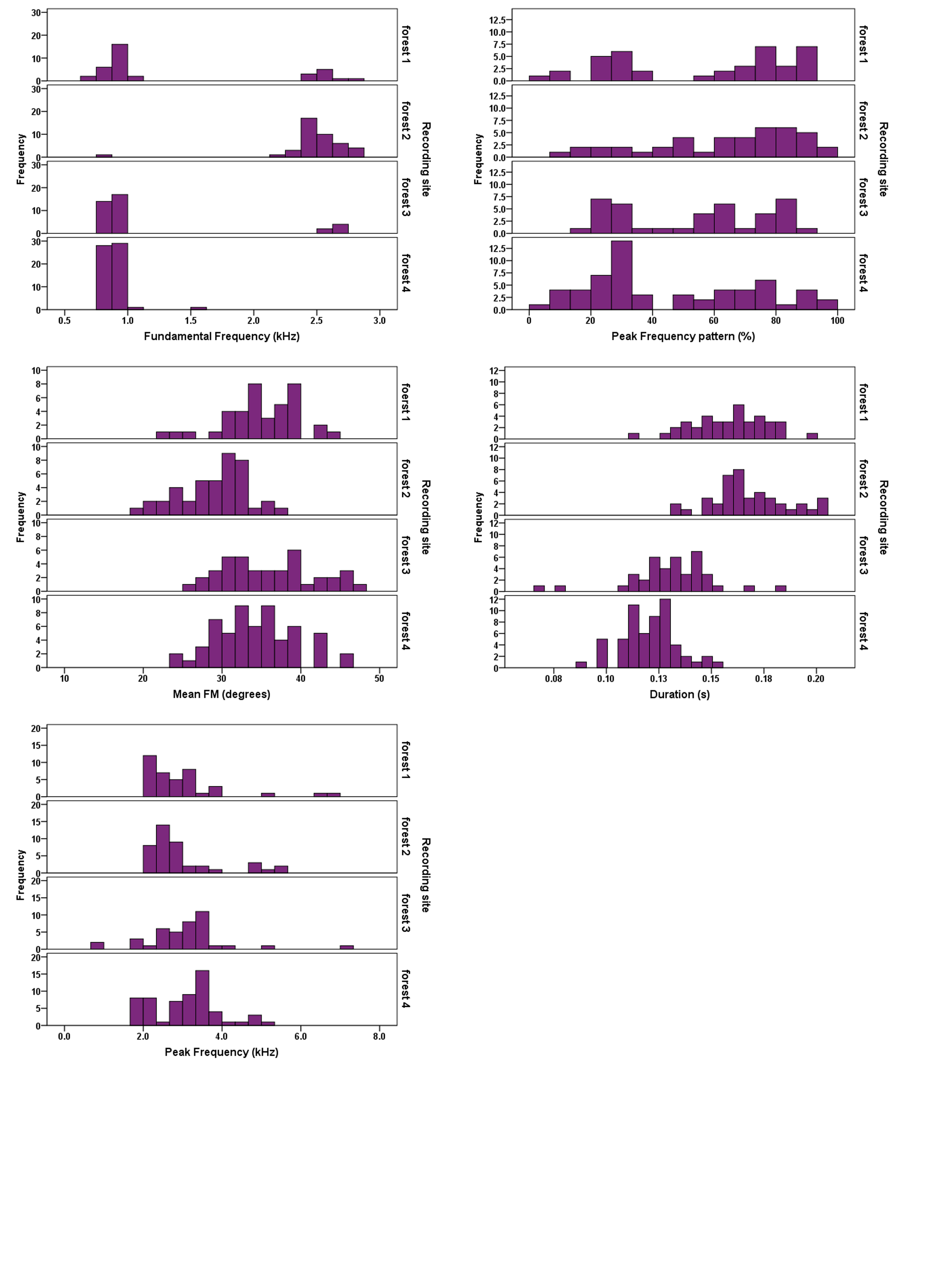

Supplement: Figure S2 — Histograms displaying the frequency distribution of values of the five acoustic variables separately for the four recording sites (Forest 1–4) comprising the area where clines in microsatellite markers and acoustic variation were observed. Each graph consists of four separate windows with histograms, each window displays the values for one recording site. Frequencies represent the number of calls. (TIF) [file pone.0050484.s002.tif]

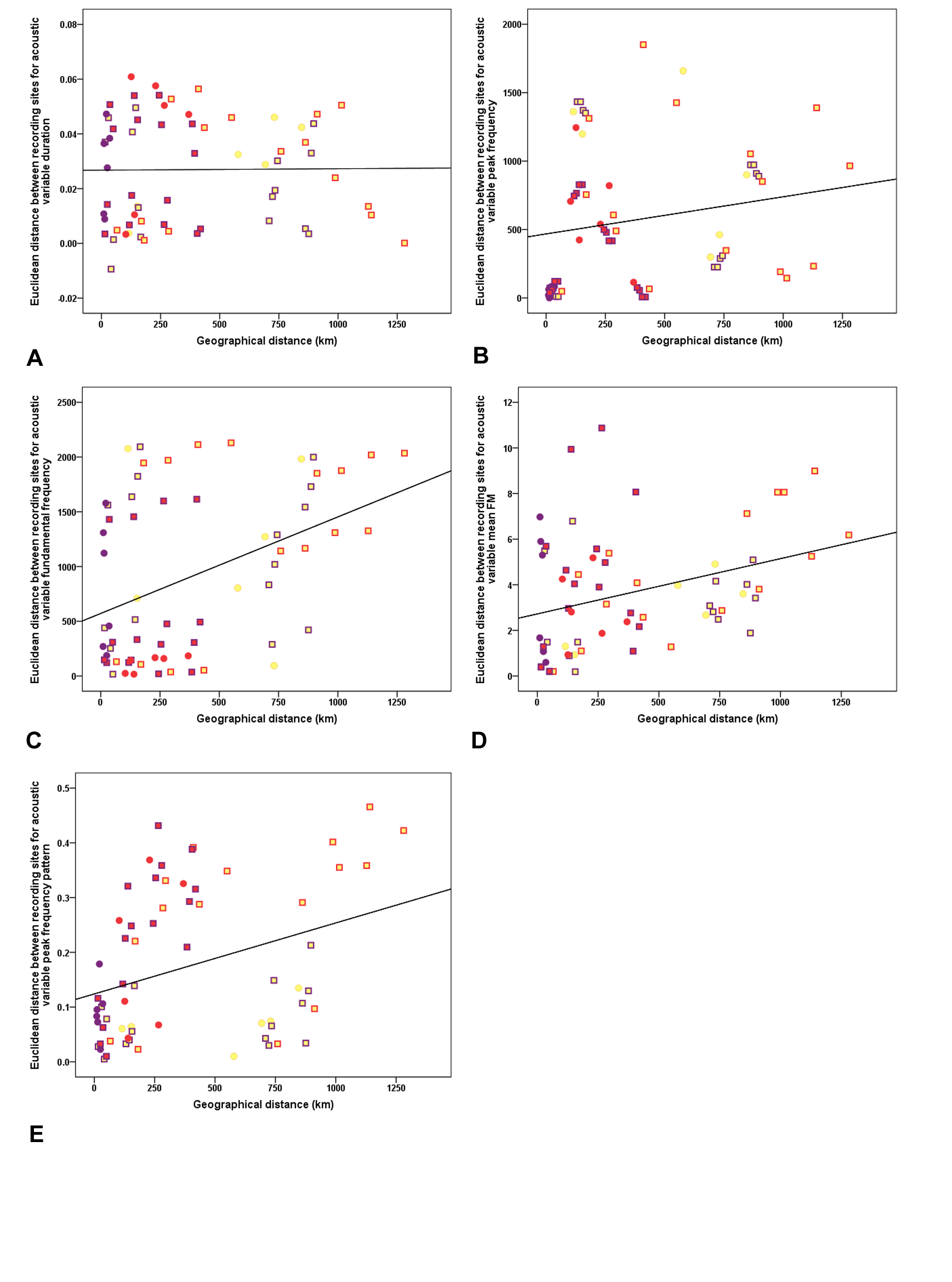

Supplement: Figure S3 — Plots of Euclidian distances between five acoustic variables derived from rosella contact calls versus geographic distance along the path of the river. The acoustic variables shown are (a) call duration, (b) peak frequency, (c) fundamental frequency, (d) mean frequency modulation, and (e) peak frequency pattern. The Euclidian distance for each acoustic variable is the absolute difference between each recording site in the average value of that acoustic variable. Circles represent comparisons among the four recording sites within each microsatellite group, and bi-coloured squares represent comparisons between recording sites in different microsatellite groups. Colours indicate the microsatellite groups involved in each comparison: Central vs. Central (yellow circles), microsatellite contact zone vs. microsatellite contact zone (purple circles), Eastern vs. Eastern (red circles), Central vs. Eastern (yellow and red squares), Central vs. microsatellite contact zone (yellow and purple squares), Eastern vs. microsatellite contact zone (red and purple squares). Mantel tests indicate significant or near-significant isolation-by-distance in fundamental frequency (r = 0.416, P = 0.033), peak frequency pattern (r = 0.338, P = 0.047), and mean frequency modulation (r = 0.338, P = 0.087), but not call duration (r = 0.010, P = 0.406) or peak frequency (r = 0.188, P = 0.173). (TIF) [file pone.0050484.s003.tif]
